# The fibrosis-plasticity axis (FPA) and ionic resilience: a systems framework for understanding adverse drug reactions in ageing populations

**DOI:** 10.3389/fphar.2026.1804876

**Published:** 2026-04-28

**Authors:** Alejandro Carballo

**Affiliations:** 1 Nässjö Läkarhus, Region Jönköping County, Nässjö, Sweden; 2 Futurum - Academy for Health and Care, Region Jönköping County, Jönköping, Sweden

**Keywords:** adaptive capacity, adverse drug reactions, ageing, fibrosis-plasticity axis, heart rate variability, ionic resilience, mechanotransduction, Na^+^/K^+^-ATPase

## Abstract

**Background:**

Older adults frequently develop disabling adverse drug reactions (ADRs) from standard-dose medications despite lacking clear organ pathology and presenting with normal serum electrolyte values. Traditional explanations focusing on altered pharmacokinetics or pharmacodynamics inadequately explain why adverse effects often span multiple organ systems and resist prediction by standard clinical parameters.

**Objective:**

To develop a unified theoretical framework—the Fibrosis-Plasticity Axis (FPA)—that integrates mechanotransduction theory, allostatic load concepts, network physiology, and electrolyte-dependent ionic resilience to explain system-level drug intolerance in ageing.

**Methods:**

We synthesize evidence from mechanobiology, ion transport physiology, autonomic function research, and geriatric pharmacology to develop an integrated framework linking tissue-level stabilization to system-level pharmacological vulnerability.

**Results:**

We identify six functional vulnerability domains where plasticity loss manifests clinically and propose that ionic resilience—the capacity to maintain electrolyte-dependent cellular functions under stress—represents a critical, yet under-measured, determinant of drug tolerance. We introduce the Composite Plasticity Index (CPI), combining dynamic capacity measures (heart rate variability, gait variability) with stabilization markers (pulse wave velocity, inflammatory markers), as a conceptual biomarker for adaptive capacity.

**Conclusion:**

The FPA framework provides mechanistic coherence for multisystem vulnerability patterns and generates testable predictions about adverse effect clustering. By shifting focus from concentration-based to capacity-based assessment, this framework may improve prediction of drug tolerance and identify targets for plasticity-restoring interventions, including the emerging paradigm of repairing without fibrosis.

## 1 Introduction

An 81-year-old woman with well-controlled hypertension and type 2 diabetes presents with profound fatigue, postural dizziness, and cognitive slowing 3 weeks after starting bisoprolol 2.5 mg daily—half the standard dose. Her blood pressure is 118/72 mmHg, heart rate 58 beats per minute, echocardiography shows preserved ejection fraction (62%), comprehensive metabolic panel reveals no abnormalities, and all electrolyte values fall within reference ranges. She has become housebound. What has failed?

This scenario exemplifies a central challenge in geriatric pharmacology: patients develop disabling symptoms from guideline-concordant therapies despite lacking identifiable pathology and presenting with normal laboratory values. Such drug intolerance affects 30%–50% of older adults on multidrug regimens yet remains poorly understood (Gurwitz et al., 2003; Budnitz et al., 2011). Traditional explanations focus on altered pharmacokinetics or pharmacodynamics, but these mechanisms inadequately explain why adverse effects often span multiple organ systems and resist prediction by standard clinical or laboratory measures (Mangoni and Jackson, 2004; McLachlan et al., 2009).

We propose an alternative: that drug intolerance in ageing populations primarily reflects progressive loss of adaptive capacity across interconnected physiological systems—conceptualized as the Fibrosis-Plasticity Axis (FPA). Within this framework, adverse drug reactions function as stress tests revealing diminished adaptive range rather than drug toxicity *per se*. Central to this adaptive capacity is ionic resilience: the ability to maintain electrolyte-dependent cellular functions under physiological and pharmacological stress.

## 2 Theoretical foundations: the fibrosis-plasticity axis

### 2.1 From molecular damage to system inflexibility

Contemporary theories of ageing emphasize accumulation—of DNA damage, protein aggregates, senescent cells, or inflammatory mediators (López-Otín et al., 2013; Franceschi et al., 2018). These frameworks have yielded major advances yet struggle to explain vulnerability phenomenology. Consider two individuals of identical age with similar comorbidities: one tolerates standard medications without difficulty; the other develops disabling side effects from minimal doses. Standard biomarkers often fail to distinguish these patients (Fried et al., 2001; Rockwood and Mitnitski, 2007).

The FPA framework complements damage-accumulation models by shifting focus from pathology to reversibility. Rather than asking “what has broken?”, we ask “what has become inflexible?” This draws on three theoretical traditions.

#### 2.1.1 Mechanotransduction and tensegrity

Ingber’s work demonstrates that cells maintain stability through balanced tension across cytoskeletal and extracellular matrix networks (Ingber, 2008; Humphrey et al., 2014). Plasticity depends on semi-stable states where mechanical forces can be redistributed dynamically. Chronic stress triggers stabilization: collagen cross-linking, myofibroblast differentiation, and loss of cytoskeletal dynamics. These changes create positive feedback where tissue stiffness activates mechanosensitive pathways (YAP/TAZ, ROCK, TGF-β), amplifying fibrotic signaling (Dupont et al., 2011; Meng et al., 2016).

#### 2.1.2 Allostatic load and regulatory exhaustion

McEwen’s framework demonstrates that chronic stress progressively narrows the range within which systems can safely vary (McEwen, 1998; Sterling, 2012). When continuous adaptation becomes unsustainable, stabilization becomes protective—trading future flexibility for immediate stability. This may explain arterial stiffening, reduced heart rate variability, and neuromuscular inflexibility observed with ageing (Zieman et al., 2005; Thayer et al., 2012).

#### 2.1.3 Network physiology and cascading vulnerability

Bartsch, Bashan, and colleagues reveal that organ systems form dynamically coupled networks where loss of flexibility in one domain propagates to others (Bartsch et al., 2015; Bashan et al., 2012). Cardiac rhythms influence respiratory patterns; autonomic tone modulates gastrointestinal function. These couplings create resilience through distributed compensation but vulnerability through cascading failure—potentially explaining why chronic disease in one system increases fragility across others (Marengoni et al., 2011; Tinetti et al., 2012).

## 3 Electrolytes and ionic resilience: the energetic foundation

### 3.1 Beyond concentration: the capacity to defend gradients

Electrolytes do not function as passive solutes. Ionic gradients across cell membranes underpin membrane potential, neuromuscular transmission, vascular tone, intracellular calcium signaling, and mitochondrial energy production (Clausen, 2003; Ingber, 2006). The Na^+^/K^+^-ATPase (sodium-potassium pump) alone accounts for approximately 19%–28% of total body ATP consumption, with even higher fractions in excitable tissues such as neurons and skeletal muscle (Pirkmajer and Chibalin, 2016; Clausen, 2013).

Skeletal muscle contains one of the largest and most dynamic pools of Na^+^/K^+^-ATPase in the body. Under resting conditions, these pumps operate at only 2%–6% of maximal capacity, but activity increases substantially during exercise or physiological stress (Clausen, 2013). The capacity for rapid upregulation of pump activity is essential for maintaining membrane excitability during repeated muscle contractions—a function critical for everyday activities and functional independence.

Evidence from both human and rodent studies demonstrates that muscle Na^+^/K^+^-ATPase content and activity decline with advancing age (Wyckelsma and McKenna, 2016; McKenna et al., 2008). This decline affects the capacity for ion regulation during stress, contributing to exercise intolerance, fatigue, and reduced functional reserve in older adults. Importantly, these changes in pump capacity occur independently of changes in resting serum electrolyte concentrations.

### 3.2 Why serum electrolyte measurements are insufficient

Routine laboratory measurements assess concentration, not capacity. This distinction has critical clinical implications.

#### 3.2.1 Compartmental mismatch

For magnesium, 98% is intracellular; only 2% circulates in serum. For potassium, less than 2% of total body content is in plasma. Intracellular depletion may precede and exist despite normal serum concentrations—a phenomenon termed ‘chronic latent deficiency’ (Barbagallo et al., 2021; Reinhart, 1988).

#### 3.2.2 Compensatory masking

Homeostatic mechanisms actively maintain serum concentrations at the expense of intracellular reserves. Bone magnesium stores buffer serum levels; muscle potassium is sacrificed to preserve plasma concentrations (Palmer, 2015).

#### 3.2.3 Static versus dynamic

A patient may present with normal serum potassium while the physiological machinery required to restore potassium homeostasis following perturbation is significantly impaired (Allen et al., 2008).

#### 3.2.4 Reference range limitations

Reference ranges reflect population distributions, not individual adequacy. A value at the lower end of normal may represent adequacy for one individual but marginal insufficiency for another with higher demands or reduced compensatory capacity.

### 3.3 The potassium-magnesium axis

Magnesium is required for Na^+^/K^+^-ATPase function; deficiency inhibits the pump, leading to intracellular potassium depletion (Swaminathan, 2003). This explains why hypokalemia is often refractory to potassium supplementation alone—magnesium repletion is required to restore cellular potassium uptake (Huang and Kuo, 2007). The Framingham Heart Study demonstrated that participants in the lowest quartile of serum magnesium had approximately 50% higher risk of developing atrial fibrillation over 20 years compared with the highest quartile—even when values fell within reference ranges (Khan et al., 2013).

## 4 Six functional vulnerability domains

**FIGURE 1:**
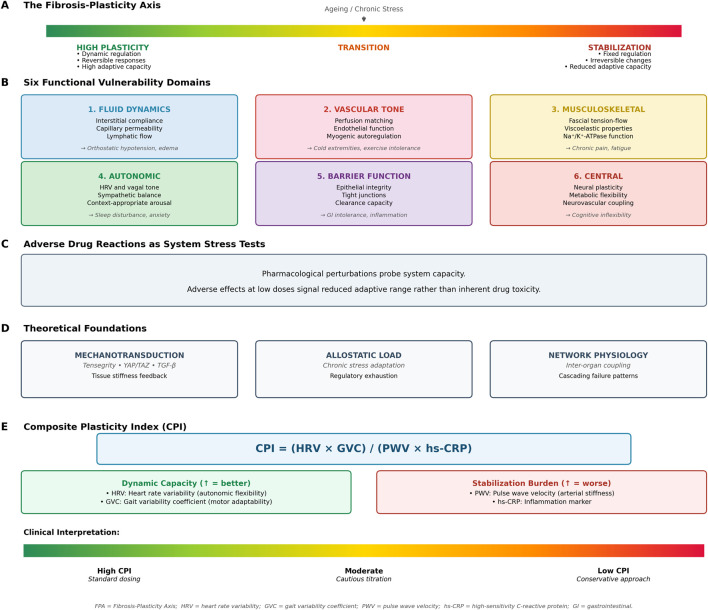
The fibrosis-plasticity axis (FPA) framework. **(A)** The fibrosis-plasticity axis. **(B)** Six functional vulnerability domains. **(C)** Adverse drug reaction as system stress test. **(D)** Theoretical foundations. **(E)** Composite Plasticity Index (CPI).

We propose six interconnected domains where plasticity loss and ionic vulnerability manifest clinically (Figure 1).

### 4.1 Domain 1: fluid dynamics and interstitial compliance

Plastic states enable rapid fluid redistribution through dynamic capillary permeability and lymphatic flow (Levick and Michel, 2010). Stabilization produces extracellular matrix stiffening and lymphatic insufficiency, creating orthostatic hypotension and dependent edema. Drugs affecting fluid balance (diuretics, ACE inhibitors, calcium antagonists, NSAIDs) produce exaggerated effects when this domain is compromised.

### 4.2 Domain 2: vascular tone and perfusion matching

Endothelial nitric oxide signaling and myogenic autoregulation enable beat-to-beat perfusion matching (Förstermann and Sessa, 2012). Arterial stiffening and endothelial dysfunction create fixed perfusion patterns with cold extremities, exercise intolerance, and postprandial hypotension.

### 4.3 Domain 3: musculoskeletal tension-flow coupling

Fascial networks exhibit viscoelastic properties distributing mechanical loads (Schleip et al., 2005; Stecco et al., 2011). Stabilization produces fascial adhesions, manifesting as chronic pain and disproportionate fatigue. Na^+^/K^+^-ATPase decline in muscle directly impairs this domain. Drugs affecting neuromuscular function (statins, corticosteroids) produce myopathy when baseline reserve is compromised.

### 4.4 Domain 4: autonomic responsiveness and rhythm

Parasympathetic-sympathetic balance generates heart rate variability and context-appropriate arousal (Thayer and Lane, 2009; Shaffer and Ginsberg, 2017). Loss of vagal tone creates fixed arousal states with sleep disturbance and anxiety. Cardiac ionic currents depend directly on electrolyte gradients. Drugs modulating autonomic tone (SSRIs, beta-blockers, anticholinergics) produce unpredictable effects.

### 4.5 Domain 5: barrier function and clearance capacity

Epithelial tight junctions and peristaltic coordination maintain selective permeability (Vancamelbeke and Vermeire, 2017). Stabilization produces altered intestinal permeability and chronic inflammation. Drugs disrupting gastrointestinal homeostasis (PPIs, antibiotics, opioids) produce exaggerated effects.

### 4.6 Domain 6: central integration and metabolic flexibility

Neural plasticity and metabolic switching provide cognitive and energetic flexibility (Citri and Malenka, 2008; Camandola and Mattson, 2017). Stabilization reduces synaptic plasticity and uncouples neurovascular responses. Neuronal Na^+^/K^+^-ATPase is essential for maintaining membrane potential and signal transmission. CNS-active agents challenge limited central adaptive capacity.

## 5 Adverse drug reactions as system stress tests

Pharmacological interventions function as controlled stressors probing system capacity. Adverse effects at low doses signal reduced adaptive range rather than inherent toxicity (Edwards and Aronson, 2000). Many commonly prescribed medications interact directly or indirectly with ionic regulation: diuretics affect renal electrolyte handling; cardiac glycosides inhibit Na^+^/K^+^-ATPase; beta-blockers modulate sympathetic tone and cellular potassium uptake; proton pump inhibitors impair magnesium absorption (Hoorn and Zietse, 2013; Perazella, 2013). A summary of common ADRs by drug class, primary FPA domain, and ionic involvement is provided in Table 1.

**TABLE 1:** Classification of common adverse drug reactions by FPA domain and ionic involvement.

Drug class	Common ADRs	Primary FPA domain	Ionic involvement
ACE inhibitors	Dizziness, fatigue, hypotension, cough	Fluid/Vascular	Volume regulation, vascular tone
Beta-blockers	Fatigue, bradycardia, cold extremities	Vascular/Autonomic	Cardiac ionic currents, K^+^ uptake
Calcium antagonists	Peripheral edema, flushing, headache	Fluid/Vascular	Ca^2+^ channels, vascular smooth muscle
Statins	Myalgia, fatigue, weakness	Musculoskeletal	Muscle membrane stability, CoQ10
SSRIs	GI disturbance, insomnia, agitation	Autonomic/Barrier	Serotonin-ion channel interactions
NSAIDs	Dyspepsia, edema, elevated BP	Barrier/Fluid	Prostaglandin-Na^+^ regulation
PPIs	Headache, GI symptoms, fatigue	Barrier/Central	Mg^2+^ absorption impairment
Metformin	GI intolerance, fatigue	Barrier/Central	Mitochondrial energetics

ADRs, adverse drug reactions; FPA, Fibrosis-Plasticity Axis; GI, gastrointestinal.

We analyzed adverse effect profiles for eight major drug classes, focusing on common (≥10%) effects. Key patterns emerged.

### 5.1 Cross-class clustering

Fatigue appeared across pharmacologically distinct classes (beta-blockers, statins, ACE inhibitors, PPIs, metformin), suggesting shared system-level mechanisms rather than drug-specific toxicity.

### 5.2 Domain-specific patterns

Drugs perturbing fluid balance consistently produced edema and hypotension; drugs affecting musculoskeletal function showed myalgia patterns; drugs modulating autonomic tone showed sleep disturbance.

### 5.3 Dose-effect nonlinearity

Clinical observations suggest that halving doses sometimes provides limited reduction in adverse effects, while quarter-doses may still produce intolerance. The FPA framework predicts this: below adaptive thresholds, perturbation magnitude matters less than remaining capacity.

## 6 Measuring adaptive capacity: the Composite Plasticity Index

If the FPA framework has validity, quantifying position along the plasticity-stabilization axis should predict drug tolerance. We propose the Composite Plasticity Index (CPI):

$$\text{CPI}=\left(\text{HRV}\times \text{GVC}\right)\;/\;\left(\text{PWV}\times \text{hs}‐\text{CRP}\right)$$

Where: HRV = heart rate variability (SDNN, ms); GVC = gait variability coefficient (%); PWV = pulse wave velocity (m/s); hs-CRP = high-sensitivity C-reactive protein (mg/L).

This dimensionless index balances dynamic capacity (numerator) against stabilization burden (denominator). Each component has established outcome associations: reduced HRV predicts cardiac events (Tsuji et al., 1996); increased gait variability predicts falls (Hausdorff et al., 2001); elevated PWV indicates arterial stiffness (Ben-Shlomo et al., 2014); hs-CRP reflects systemic inflammation (Ridker et al., 2017). The CPI requires prospective validation before clinical application.

The rationale parallels that of HOMA-IR for insulin resistance: just as fasting glucose may be normal in individuals with significant insulin resistance, serum electrolytes may be normal in individuals with significantly impaired ionic resilience. The CPI attempts to capture functional capacity rather than static concentration (Matthews et al., 1985; Wallace et al., 2004). Table 2 contrasts the CPI with conventional laboratory assessment and frailty indices across key dimensions.

**TABLE 2:** Comparison of assessment approaches for pharmacological vulnerability.

Dimension	Laboratory values	Frailty index	CPI (FPA framework)
Primary question	Within reference range?	How many deficits?	How much capacity remains?
Focus	Static concentration	Accumulated deficits	Functional adaptive capacity
Time sensitivity	Point-in-time	Low (stable)	High (dynamic)
Reversibility	Yes (supplementation)	Limited	Potential (intervention target)
Mechanistic anchoring	Strong (specific ions)	Weak (phenotypic)	Strong (ionic/energetic)
ADR prediction	Weak (if normal)	Modest	Hypothesized: High

CPI, composite plasticity index; FPA, Fibrosis-Plasticity Axis; ADR, adverse drug reaction.

## 7 Therapeutic implications

If drug intolerance reflects lost adaptive capacity, interventions restoring plasticity may improve medication tolerance.

### 7.1 Exercise

Physical activity opposes stabilization through mechanotransduction-stimulated matrix remodeling, mitochondrial biogenesis, and anti-inflammatory effects (Booth et al., 2012). Exercise also increases muscle Na^+^/K^+^-ATPase content and activity, directly improving ionic resilience (Clausen, 2003).

### 7.2 Deprescribing

Systematic medication review reduces cumulative system stress, potentially allowing adaptive range recovery (Scott et al., 2015; Page et al., 2016). Reduced drug burden may allow recovery of tolerance to necessary therapies.

### 7.3 Electrolyte optimization

In patients with borderline values—especially those with patterns of pharmacological intolerance—empirical magnesium and potassium supplementation may restore ionic reserve even when values fall within reference ranges.

### 7.4 Heart rate variability biofeedback

Breathing exercises can increase vagal tone and may restore aspects of autonomic flexibility (Lehrer and Gevirtz, 2014).

The framework also suggests novel targets: matrix metalloproteinase modulators for enhanced remodeling (Bonnans et al., 2014); strategies supporting endothelial glycocalyx function (Chappell et al., 2014); NAD + precursors for mitochondrial support (Rajman et al., 2018); and senolytic therapies (Kirkland and Tchkonia, 2020). These require rigorous testing before clinical application.

### 7.5 Repairing without fibrosis: a therapeutic paradigm

The interventions described above share a common logic that deserves explicit articulation: they aim not to suppress symptoms or compensate for lost function, but to restore the physiological conditions under which adaptive responses become possible again. We term this approach repairing without fibrosis—a therapeutic orientation that targets the stabilization-plasticity balance itself rather than individual pathological endpoints.

This paradigm rests on a critical biological premise: that fibrotic stabilization, while progressive, is not uniformly irreversible. Mechanobiological evidence suggests that early-to-intermediate stabilization states retain substantial remodeling potential. Matrix metalloproteinases can degrade pathological collagen cross-links when upstream mechanotransduction signals shift toward plasticity-favoring states (Bonnans et al., 2014). YAP/TAZ activity, which amplifies fibrotic signaling under mechanical rigidity, can be modulated by reducing substrate stiffness or restoring cytoskeletal dynamics through physical activity (Dupont et al., 2011). Senolytic clearance of SASP-generating cells removes a key source of chronic TGF-β signaling that maintains fibrotic tone (Kirkland and Tchkonia, 2020).

The clinical implication is that patients identified as pharmacologically vulnerable by low CPI scores are, by the same logic, primary candidates for plasticity-restoring intervention. Rather than accepting stabilization as an irreversible feature of biological ageing, the FPA framework reframes it as a potentially modifiable state—at least within a therapeutic window that likely narrows with advancing age and cumulative allostatic burden.

This reorientation has practical consequences for how geriatric care is structured. Current paradigms optimize drug selection for a fixed physiological substrate. A plasticity-restoration paradigm would instead attempt to expand the substrate itself—improving drug tolerance as a downstream consequence of restored adaptive capacity. Whether this window is sufficiently wide to be clinically meaningful remains an empirical question, one that the proposed validation studies (Table 3) are designed in part to address.

**TABLE 3:** Proposed clinical studies to validate the FPA framework.

Study	Design	N/Duration	Primary outcome
CPI-guided prescribing	RCT	600/12 months	ADRs requiring dose reduction or discontinuation
Plasticity restoration	RCT	400/6 months	Medication tolerance at 6 months
Ionic resilience validation	Observational	300/Cross-sectional	Correlation: CPI components vs. drug tolerance
Domain clustering	Retrospective cohort	10,000+/Database	ADR clustering by FPA domain vs. drug class

CPI, composite plasticity index; RCT, randomized controlled trial; ADR, adverse drug reaction.

## 8 Research agenda and testable predictions

**FIGURE 2:**
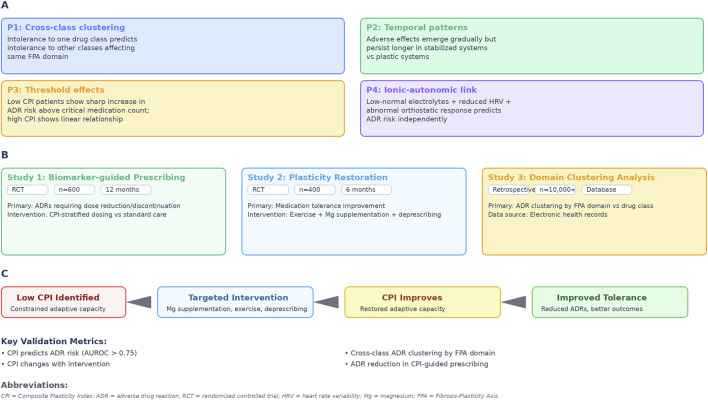
Testable predictions and validation framework. **(A)** Testable predictions from the FPA framework. **(B)** Proposed validation studies. **(C)** Expected outcomes if framework valid. Abbreviations: CPI, composite plasticity index; ADR, adverse drug reaction; RCT, randomized controlled trial; HRV, heart rate variability; MG, magnesium; FPA, fibrosis-plasticity axis.

The FPA framework generates falsifiable predictions (Figure 2):

**Prediction 1:** Individuals intolerant to one drug class should show elevated risk for pharmacologically distinct classes affecting similar FPA domains (cross-class clustering by domain).
**Prediction 2:** Adverse effects should emerge gradually but persist longer in stabilized systems compared to plastic systems (temporal pattern analysis).
**Prediction 3:** In individuals with low CPI, adverse effect risk should increase sharply above critical medication counts, whereas those with preserved CPI show more linear relationships (threshold effects).
**Prediction 4:** Low-normal electrolyte values combined with reduced HRV and abnormal orthostatic responses should predict ADR risk independently of serum concentrations alone.

## 9 Limitations

This framework has important limitations. The FPA framework remains theoretical pending empirical validation; alternative conceptual organizations may prove superior. The six domains are heuristic categories with substantial overlap. Not all vulnerability can be explained through lost adaptive capacity—genetic factors, idiosyncratic reactions, and specific pathological processes remain important.

Current biomarkers capture only aspects of adaptive capacity. The proposed CPI requires validation in diverse populations and will likely need refinement. Correlation between adaptive capacity measures and drug intolerance does not establish causation. The degree to which adaptive capacity can be restored at different ageing stages remains unclear—there may be inflection points beyond which stabilization becomes largely irreversible.

## 10 Conclusion

The Fibrosis-Plasticity Axis provides a conceptual framework for understanding why older adults develop disabling adverse drug reactions from standard medications despite lacking clear pathology and presenting with normal laboratory values. By integrating mechanotransduction theory with ionic resilience, we propose that drug intolerance primarily reflects diminished adaptive capacity rather than drug toxicity *per se*.

The distinction between concentration and capacity is not merely semantic—it has direct implications for clinical decision-making. A patient with normal serum electrolytes but constrained ionic resilience may warrant the same pharmacological caution as one with overt laboratory abnormalities. The framework generates testable predictions and identifies intervention targets, potentially shifting prescribing from age-based approaches toward adaptive capacity-guided personalization.

Crucially, the framework also identifies a therapeutic horizon beyond prediction: the possibility of repairing without fibrosis. If plasticity loss drives pharmacological vulnerability, then interventions that restore tissue and systemic plasticity—through mechanical loading, ionic repletion, senolytic therapy, or deprescribing—represent not merely supportive measures but potentially transformative ones. The ultimate measure of theoretical frameworks in medicine is not their elegance but their usefulness—whether they improve prediction, guide intervention, or reveal hidden patterns. We believe the Fibrosis-Plasticity Axis warrants empirical investigation and may offer practical value for clinicians caring for vulnerable older adults. The evidence will decide.

## Data Availability

The original contributions presented in the study are included in the article/supplementary material, further inquiries can be directed to the corresponding author.
